# Thermally Stable Ceramic-Salt Electrolytes for Li Metal Batteries Produced from Cold Sintering Using DMF/Water Mixture Solvents

**DOI:** 10.3390/nano13172436

**Published:** 2023-08-28

**Authors:** Sunwoo Kim, Yejin Gim, Wonho Lee

**Affiliations:** 1 Department of Polymer Science and Engineering, Kumoh National Institute of Technology, Gumi 39177, Republic of Korea; 2 Department of Energy Engineering Convergence, Kumoh National Institute of Technology, Gumi 39177, Republic of Korea

**Keywords:** electrolyte, ionic conductivity, low temperature, lithium metal batteries

## Abstract

The cold sintering process (CSP) for synthesizing oxide-based electrolytes, which uses water transient solvents and uniaxial pressure, is a promising alternative to the conventional high temperature sintering process due to its low temperature (<200 °C) and short processing time (<2 h). However, the formation of amorphous secondary phases in the intergranular regions, which results in poor ionic conductivity (σ), remains a challenge. In this study, we introduced high-boiling solvents of dimethylformamide (DMF, b.p.: 153 °C) and dimethyl sulfoxide (DMSO, b.p.: 189 °C) as transient solvents to develop composite electrolytes of Li_1_._5_Al_0_._5_Ge_1_._5_(PO_4_)_3_ (LAGP) with bis(trifluoromethane)sulfonimide lithium salt (LiTFSI). Our results show that composite electrolytes processed with the DMF/water mixture (CSP LAGP-LiTFSI DMF/H_2_O) yield a high σ of 10^−4^ S cm^−1^ at room temperature and high relative densities of >87%. Furthermore, the composite electrolytes exhibit good thermal stability; the σ maintains its initial value after heat treatment. In contrast, the composite electrolytes processed with the DMSO/water mixture and water alone show thermal degradation. The CSP LAGP-LiTFSI DMF/H_2_O composite electrolytes exhibit long-term stability, showing no signs of short circuiting after 350 h at 0.1 mAh cm^−2^ in Li symmetric cells. Our work highlights the importance of selecting appropriate transient solvents for producing efficient and stable composite electrolytes using CSP.

## 1. Introduction

The increasing demand for electronic devices and electric vehicles (EVs) is driving the need for Li-ion batteries with higher energy densities and longer lifespans [1,2,3,4,5,6]. However, the use of graphite anodes in commercial Li-ion batteries restricts their energy densities due to their low theoretical capacity (372 mAh g^−1^) of graphite, making them heavy and unable to meet the demands of EVs, which require lighter electronic devices and the capacity for longer driving distances. To overcome this limitation, Li metal is being explored as a next-generation anode, due to its low electrochemical potential (−3.04 V vs. SHE) and high specific capacity (3860 mAh g^−1^), which is ten times greater than that of graphite [2,7,8,9,10,11,12,13]. However, the use of liquid electrolytes in Li metal batteries poses a challenge due to flammable explosions caused by Li dendrite growth and short circuits [14,15,16,17,18,19,20,21,22].

The development of solid electrolytes with both high electrochemical stability and non-flammability is crucial for advancing Li metal batteries [8,17,18,19,20,21,22,23,24,25,26,27,28]. Among the various solid electrolytes, oxide-based ones show potential due to their mechanical strength and electrochemical stability, which can mitigate the safety concern associated with Li dendrite growth [23,25,26,27,28,29]. NASICON-type ceramic electrolytes, in particular, have gained attention due to their high ionic conductivity (σ) of approximately 10^−4^ S cm^−1^ at room temperature [30], stability against water and oxygen, and a Li transfer number close to 1 [31]. However, producing solid electrolytes using these oxide ceramics is challenging, as it requires a harsh process involving high-temperature sintering above 800 °C for a long period of time, therefore, it is both time-consuming and expensive. Furthermore, this high-temperature process can reduce the σ via the evaporation of Li from the ceramic electrolyte [32]. To address these issues, researchers are exploring alternative processing methods, such as ultra-high temperature sintering, flash sintering, photonic sintering, the cold sintering process (CSP), etc. [33,34,35,36].

CSP is an attractive alternative to high-temperature sintering for producing oxide-based solid electrolytes, as it can take place at very low temperatures (100~200 °C) over short durations (less than 2 h), and it is assisted by a small amount of water and uniaxial pressure. During CSP, the ceramic particles melt and precipitate to form an amorphous phase [36,37,38,39,40,41]. Therefore, this process has been reported to result in a poor σ compared with conventionally sintered ceramic electrolytes [42,43] due to the slightly low relative densities (e.g., low densification) and the formation of amorphous phase along the grain boundary [32]. To solve these problems, researchers have been exploring the alternative CSP method, which uses transient solvents other than water, or they have introduced second additives. For example, Sun’s group introduced 1 M acetic acid-DMSO as CSP transient solvents instead of water in order to produce Li_1_._3_Al_0_._3_Ti_1_._7_(PO_4_)_3_ (LATP) electrolytes; the results showed a higher relative density of 93% and an increased σ of 8.04 × 10^−5^ S cm^−1^ compared with those found for CSP LATP using water solvents (2.79 × 10^−5^ S cm^−1^) [44]. To mitigate sluggish ionic conduction in the amorphous grain boundaries, Lu’s group prepared Li_1_._5_Al_0_._5_Ge_1_._5_(PO_4_)_3_ (LAGP) electrolytes by incorporating lithium perchlorate (LiClO_4_) at a low temperature of 120 °C. They observed that the σ and activation energy of the electrolyte were enhanced from 3.8 × 10^−5^ S cm^−1^ and 0.38 eV to 6.4 × 10^−5^ S cm^−1^ and 0.26 eV, respectively [45]. Similarly, Gomez’s group showed that cold-sintered LAGP and LATP can achieve high σ at an order of 10^−4^ S cm^−1^ by introducing bis(trifluoromethane)sulfonimide lithium salt (LiTFSI) [46]. They found out that the water-in-salt phase formed along the grain boundary, after completing the CSP of LAGP (or LATP), greatly improves Li ion conduction. Despite their success in terms of improved σ, the water-in-salt phase located at the grain boundary of the oxide electrolytes may cause the degradation of Li metal and evaporation of water when exposed to heat, presenting a serious challenge to this approach [47].

We propose a lost-cost synthetic approach for producing thermally stable, highly conductive NASICON-type solid electrolytes. We selected LAGP as the main ionic conductor and LiTFSI as an additive to produce a CSP LAGP–LiTFSI electrolyte. To address the issues associated with the typical transient solvents of water for CSP, we introduced the following high-boiling point solvents as CSP processing solvents: *N*,*N*-dimethylformamide (DMF, b.p.: 153 °C) or dimethyl sulfoxide (DMSO, b.p.: 189 °C). Our results demonstrate that using a DMF/H_2_O mixture is beneficial for synthesizing thermally stable, highly conductive CSP LAGP–LiTFSI, whereas using a DMSO/H_2_O mixture and water alone leads to poor thermal stability. Furthermore, the CSP LAGP–LiTFSI DMF/H_2_O composite electrolytes exhibit long-term stability, showing no signs of short circuiting until 350 h at 0.1 mAh cm^−2^ in Li symmetric cells. Our work extends the operating temperature of the solid electrolytes processed by CSP, making it suitable for charging and discharging Li ion batteries under harsh conditions.

## 2. Materials and Methods

### 2.1. Materials

LAGP powder was purchased from MSE supplies LLC. LiTFSI and LiTFI were purchased from Sigma Aldrich (St. Louis, MO, USA) and TCI (Tokyo, Japan), respectively. Lithium foil (13 mm diameter × 170 μm thickness) was purchased from the MTI Corporation (Richmond, CA, USA). DMSO and DMF were purchased from Daejung (Siheung-si, Republic of Korea).

### 2.2. Preparation of Electrolytes Using CSP

To prepare CSP LAGP–LiTFSI DMF/H_2_O, DMF and H_2_O solvents were mixed in various volume ratios, from 1:9 to 7:3, using a LiTFSI with a 0.89 to 6.14 M concentration. Then, the solution was mixed with LAGP powder using an agate mortar and pestle. The moistened powder was cold sintered based on a procedure in the literature [46]; all the samples were heated at 150 °C for 1 h. The prepared pellets were polished using 400, 1500, and 3000 grit sandpaper to remove impurities. For the preparation of the CSP LAGP–LiTFSI DMSO/H_2_O, all processes were the same, except the use of DMSO and H_2_O as transient solvents. For comparison, CSP LAGP–LiTFSI was also prepared by using only H_2_O as a solvent. All of the prepared pellets were round in shape with a thickness of about 1 mm and a diameter of 13 mm. It was assumed that the LiTFSI in the solution was incorporated into the ceramic electrolytes during CSP.

### 2.3. Characterization

Relative densities were calculated based on a procedure in the literature [46]. σ values were obtained using WizEIS-1200 Premium (Daejeon-si, Republic of Korea), with an AC amplitude of 10 mV in the range of 10^−1^–10^5^ Hz. Au (100 nm) was used for the electrodes, and it was coated on both sides of the electrolytes using a Cressington sputter coater 108. Microstructures of the fractured surfaces of electrolytes were obtained using a field emission scanning electron microscope (FESEM, MAIA III) (Brno, Czech Republic). Crystal structures and phase purity were determined via X-ray diffraction (XRD, SWXD (D-MAX/2500-PC)) (Tokyo, Japan) and Cu Kα radiation in the 2θ range of 10°–70°. ^1^H NMR (Bruker, AVANCE III 400 MHz) (Billeriaca, MA, USA) spectra were acquired, using CDCl_3_ as a solvent. The symmetric coin cells, with a configuration of Li/electrolytes/Li, were assembled in an argon-filled glovebox; to reduce area specific resistance (ASR), the thicknesses of the electrolytes were reduced to 800 μm by polishing them. In addition, a small amount of 4 M LiFSI solution in dimethoxyethane (DME) (5.0 μL cm^−2^) was added in-between the Li metal and electrolytes to minimize interfacial resistance. The galvanostatic Li^+^ charge and discharge measurements of the Li/electrolytes/Li cells were measured using a Neware Battery Testing System CT-4000 (Kowloon Bay, Hong Kong) at room temperature.

## 3. Results and Discussion

**Figure 1:**
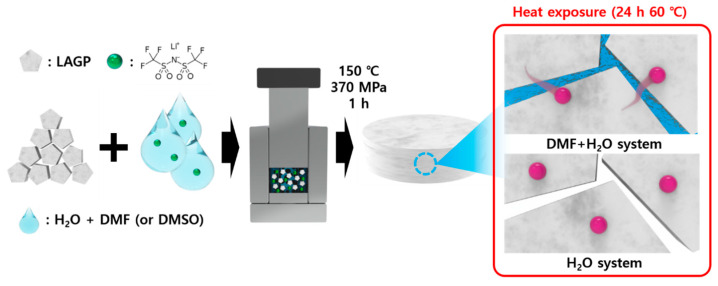
Illustration of CSP at 150 °C using co-solvents (DMF (or DMSO)/H_2_O) to produce ceramic-salt composite electrolytes.

Figure 1 shows the overall CSP process of the ceramic-salt composite electrolytes using co-solvents of DMF/H_2_O or DMSO/H_2_O; we chose high-boiling point solvents of DMF (b.p 153 °C) and DMSO (b.p 189 °C) as the second CSP solvent to produce thermally stable and solid electrolytes. In order to obtain high conductivity for the LAGP electrolytes, we reduced grain boundary resistance, and we introduced LiTFSI into CSP solvents, in accordance with a previous report [46].

**Figure 2:**
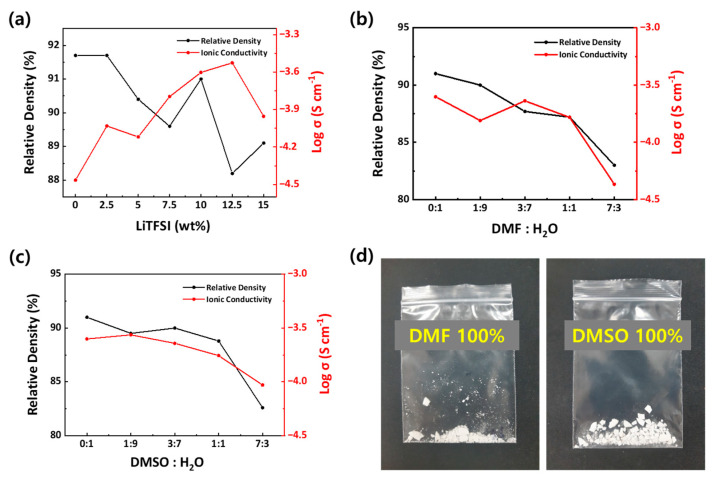
Relative densities and σ values at room temperature of (**a**) CSP LAGP–LiTFSI H_2_O composite electrolytes with different LiTFSI contents, (**b**) CSP LAGP–LiTFSI DMF/H_2_O and (**c**) CSP LAGP–LiTFSI DMSO/H_2_O. (**d**) Photograph of cold-sintered CSP LAGP–LiTFSI which only used DMF and DMSO.

First, we optimized the ceramic-salt composite electrolytes by adding different LiTFSI contents, following the procedure described previously in the literature [46]. All the electrolytes were synthesized with CSP, using water as transient solvent under a 370 MPa at 150 °C for 1 h; it is notable that CSP can take place at low temperatures and over short durations when assisted by aqueous solvents and uniaxial pressure [37,38,39]. Figure 2a shows the relative densities and total σ of the CSP LAGP–LiTFSI H_2_O containing different contents of LiTFSI. A detailed procedure for obtaining relative densities and σ is described in the Experimental Section. We also provide the representative plots of EIS measurements to obtain σ values in Figure S1. In the absence of LiTFSI, we successfully synthesized CSP LAGP electrolytes with a high relative density of approximately 91.7%. When LiTFSI was added to produce CSP LAGP–LiTFSI, σ values were gradually increased and the highest σ of 3 × 10^−4^ S cm^−1^ was obtained with 12.5 wt% LiTFSI; this is because of the formation of the ionic conduction phase involving LiTFSI water along the grain boundary of LAGP [46]. We attributed the reduction in σ, and the excess amount of LiTFSI (15 wt%), to the strong interaction between the anions and cations and the formation of the highly viscous phase at grain boundary [48]. In the case of relative densities, values were constant, at approximately 90%, in a range of 2.5 to 10 wt% LiTFSI, but they dropped slightly below 89% at 12.5 and 15 wt% LiTFSI. Overall, the trend of σ and relative densities with different LiTFSI contents is similar to a previous report [46].

Next, based on the optimized LiTFSI contents (e.g., 10 wt% LiTFSI) that comprised both high σ and relative densities, we cold sintered LAGP–LiTFSI electrolytes using a co-solvent system of DMF/H_2_O and DMSO/H_2_O. Relative density and σ values, with different DMF (or DMSO) and H_2_O ratios, are plotted in Figure 2b,c, respectively, and their values are summarized in Table S1. As shown in Figure 2b, the relative densities of CSP LAGP–LiTFSI DMF/H_2_O tend to gradually decrease with increasing DMF ratio, and it falls below 85% at a DMF:H_2_O = 7:3 ratio, which might be due to the poor dissolution ability of DMF for LAGP used in this study [38]. However, the σ remained similar with regard to the 0:1 to 1:1 ratios, showing a high conductivity of 1.65 × 10^−4^ S cm^−1^ at 1:1. With the excess amount of DMF (7:3 ratio), the σ rapidly decreases because LAGP electrolytes are not effectively cold sintered at a low relative density of 83%. CSP LAGP–LiTFSI DMSO/H_2_O also demonstrates a similar trend with CSP LAGP–LiTFSI DMF/H_2_O (Figure 2c). They have high σ at an order of 10^−4^ S cm^−1^, with 0:1 to 1:1 ratios, and σ was decreased to ~10^−5^ S cm^−1^ at a 7:3 ratio. In order to elucidate why the densification of CSP LAGP–LiTFSI does not effectively occur with an excess amount of organic solvents, such as DMF and DMSO, we also tried to cold sinter LAGP–LiTFSI using only DMF or DMSO without H_2_O. As shown in Figure 2d, we were not able to produce densified LAGP pellets, but we did obtain LAGP in a powdered form. Therefore, we conclude that water is essential for CSP, and the CSP of LAGP electrolytes needs at least a 30% water content in co-solvent systems [36].

In order to demonstrate how different processing solvents affect the crystal structures of LAGP, we measured XRD for the LAGP powder, and CSP LAGP–LiTFSI electrolytes were prepared with different solvents (Figure S2); from this point, we selected CSP LAGP–LiTFSI electrolytes synthesized using DMF (or DMSO):H_2_O = 1:1 ratios for the representative samples since they exhibit an excellent performance in terms of relative densities, σ, and thermal stability, which will be discussed later. In accordance with previous studies, the main phase of NASICON-type hexagonal structures was observed for all the samples, including the LAGP powder and CSP LAGP–LiTFSI electrolytes, and no new impurity peak was produced after CSP [46,49]. This means that the use of DMF and DMSO solvents for CSP does not affect the crystalline structure of LAGP.

**Figure 3:**
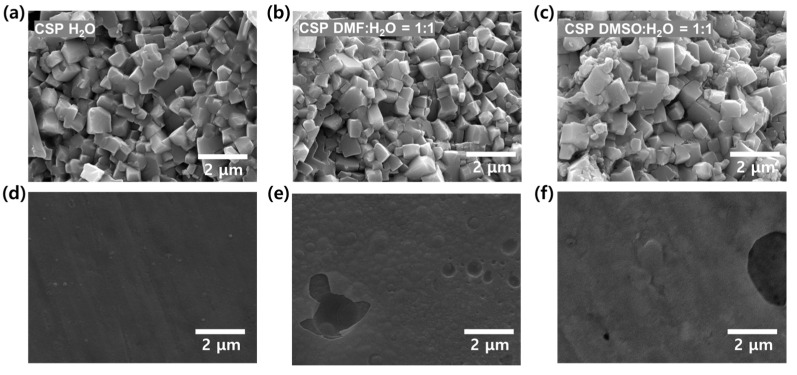
SEM images of (**a**–**c**) the fractured surfaces and (**d**–**f**) polished surfaces of CSP LAGP–LiTFSI electrolytes.

Then, the densified microstructure of CSP LAGP–LiTFSI, with or without DMF and DMSO, was characterized using SEM. We present the fractured surfaces and polished surfaces of CSP LAGP–LiTFSI electrolytes in Figure 3a–c and Figure 3d–f, respectively. In the fractured surface images, ceramic particles were effectively densified without noticeable pore and grain growth effects, and this occurred in all of the CSP LAGP–LiTFSI samples [50]. In accordance with the data concerning relative densities in Figure 2b,c, introducing 50 vol% DMF (or DMSO) does have adverse effects on the densification of LAGP via CSP. However, after examining the surface images after polishing the fractured surfaces of CSP LAGP–LiTFSI, it is evident that the samples of CSP DMF (or DMSO):H_2_O = 1:1 exhibited slightly rougher surfaces, including holes, as compared with that of CSP H_2_O (Figure 3d–f). Pure water solvents have the obvious advantage of obtaining highly densified CSP LAGP–LiTFSI, but it has a significant drawback that is discussed in the next paragraph.

**Figure 4:**
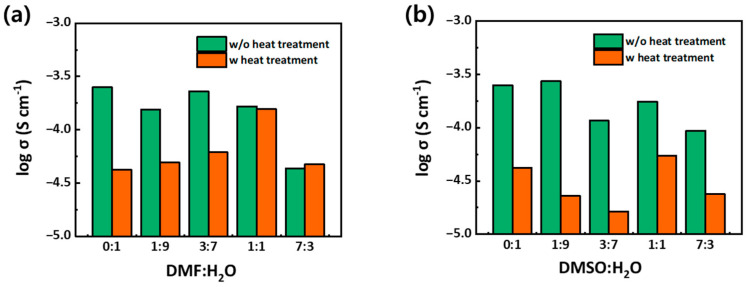
σ values of (**a**) CSP LAGP–LiTFSI DMF/H_2_O and (**b**) CSP LAGP–LiTFSI DMSO/H_2_O with and without heat treatment; the heat treatment was conducted at 60 °C for 24 h.

We compared the thermal stabilities of CSP LAGP–LiTFSI H_2_O, CSP LAGP–LiTFSI DMF/H_2_O, and CSP LAGP–LiTFSI DMF/H_2_O by exploring changes in σ behavior under heat treatment. The as-prepared electrolytes were exposed to heat 60 °C for 24 h before taking EIS measurements. We plotted the σ of CSP LAGP–LiTFSI, with and without heat treatment, by varying the DMF (or DMSO):H_2_O ratios (Figure 4). As shown in Figure 4a, the σ of CSP LAGP–LiTFSI using water alone greatly decreased after heat treatment. This is because water molecules in the “water-in-salt” phase, which is distributed along the grain boundary of CSP LAGP–LiTFSI, evaporate with heat treatment, and thus, the resulting CSP LAGP–LiTFSI loses its ion conduction channels [46]. When increasing the amount of DMF, the σ values after heat treatment were gradually improved. Then, interestingly, when the ratio of DMF:H_2_O reached 1:1, the σ with heat treatment retained its initial σ value. We speculate that CSP LAGP–LiTFSI DMF/H_2_O with 1:1 ratio maintains the DMF–LiTFSI ion conduction phase because DMF molecules do not easily evaporate due to the high b.p. of DMF (153 °C) [24,51]. On the other hand, although the DMSO solvent also has a high b.p. of 189 °C, which is even higher than that of DMF, we observed a different behavior under heat treatment (Figure 4b). CSP LAGP–LiTFSI DMSO/H_2_O does not show any thermally stable properties; the σ values for all ratios decreased after heat treatment.

**Figure 5:**
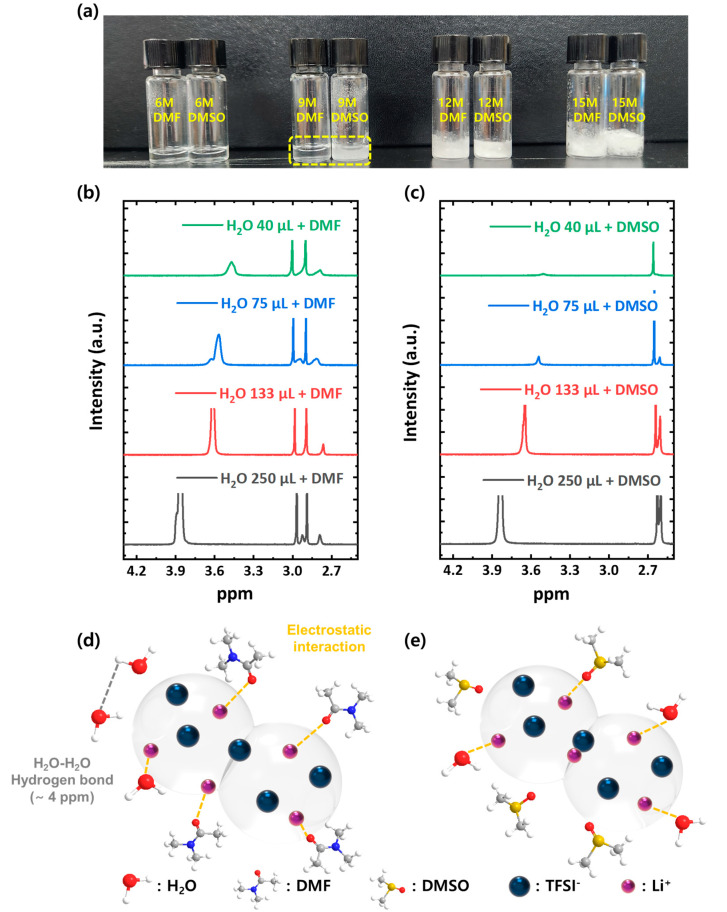
(**a**) The photograph showing DMF– and DMSO–LiTFSI solutions at different concentration. ^1^H NMR spectra of (**b**) DMF/H_2_O-LiTFSI and (**c**) DMSO/H_2_O-LiTFSI with different H_2_O contents; the amount of solvent (DMF and DMSO) and LiTFSI was fixed to 100 μL and 0.6 g, respectively. Illustration of molecular interactions between the (**d**) DMF-H_2_O-LiTFSI and (**e**) DMSO-H_2_O-LiTFSI solutions.

During the CSP, densification takes place as the aqueous solvents evaporate [50,52]. As discussed above, the phase formed by residual solvent-LiTFSI along the grain boundary of LAGP plays a critical role in achieving a high σ; we speculate that the low b.p. water evaporates first, and a small amount of high b.p. DMF and DMSO remain in the grain boundary after CSP is complete, which produces a highly concentrated DMF (or DMSO)–LiTFSI phase, and it enables fast Li ion conduction at the grain boundary of LAGP. Based on this mechanism, we attribute the better thermal stability of CSP LAGP–LiTFSI DMF/H_2_O, compared with CSP LAGP–LiTFSI DMSO/H_2_O, to the strong solvation ability of DMF [53,54]. To verify the hypothesis, we simply measured the solubility of LiTFSI in DMF and DMSO solvents by preparing highly concentrated LiTFSI solutions (5~16 M conc.) at room temperature. As shown in Figure 5a, different solubility levels of DMF and DMSO were clearly observed; DMF can completely dissolve LiTFSI up to 9 M, but only 6 M conc. of DMSO–LiTFSI solution can be made. This result indicates that DMF has a stronger solvation ability than DMSO.

Next, in order to clearly elucidate the different solvation ability of DMF and DMSO, we used ^1^H NMR of DMF– and DMSO–LiTFSI with varying amounts of water. To mimic our experimental conditions for producing CSP LAGP–LiTFSI DMF/H_2_O and CSP LAGP–LiTFSI DMSO/H_2_O, we prepared DMF– and DMSO–LiTFSI solutions with a fixed conc. of 6 g mL^−1^, and we made four ^1^H NMR samples by gradually reducing the amount of water from 250 μL to 40 μL. The complete ^1^H NMR spectra are displayed in Figure S3. The ^1^H peaks found in the 3.5~4.0 ppm range are due to the hydrogen-bonded H_2_O molecules, which are shown separately in Figure 5b,c [55,56]. As the H_2_O content in DMF/H_2_O-LiTFSI decreased, the hydrogen-bonded proton peaks shifted upwards, and they showed decreased peak intensities, indicating a decrease in the hydrogen bond strength of water molecules. This is because water molecules started to participate in solvating LiTFSI (reduced free water molecules) as the amount of water decreased. The same trend was observed for DMSO/H_2_O–LiTFSI, but with a key difference; the proton peak almost disappeared with a small amount of water (40 μL). This implies that most of the water molecules were interacting with LiTFSI, and no free hydrogen-bonded H_2_O was present. The situation is depicted in Figure 5d. In the case of DMF/H_2_O–LiTFSI, DMF has a high solvation ability for LiTFSI; thus, H_2_O–H_2_O hydrogen bonds can exist in spite of a very low H_2_O content. On the contrary, in the case of DMSO/H_2_O–LiTFSI, due to DMSO’s low solvation ability, the number of water molecules participating in solvating LiTFSI is much higher than that of DMF/H_2_O-LiTFSI. As a result, no free hydrogen-bonded water molecules are observed. This experiment demonstrates the higher solvation capacity of DMF solvents compared with DMSO.

**Figure 6:**
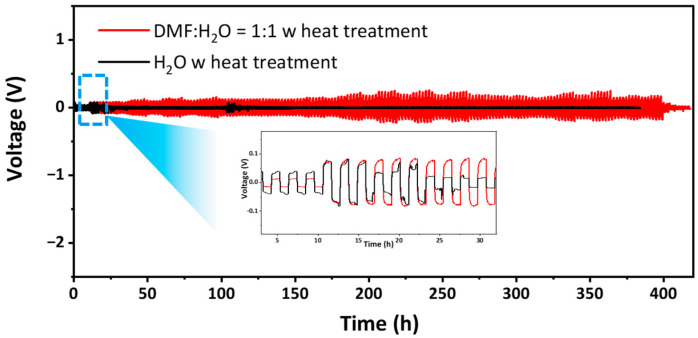
Polarization plots of the Li|CSP LAGP–LiTFSI DMF/H_2_O|Li cell and Li|CSP LAGP–LiTFSI H_2_O|Li cell at 25 °C, with current densities of 0.05 mAh cm^−2^ and 0.1 mAh cm^−2^, respectively. Both electrolytes were assembled after heat treatment.

To examine the effect of a change in the σ of composite electrolytes after heat treatment on the charge–discharge cycle, we fabricated Li symmetric cells using heat-treated composite electrolytes synthesized with DMF:H_2_O (1:1) and pure H_2_O transient solvents, as described in the Experimental Section. We noted that in a previous report, a small amount of liquid electrolyte (4 M LiFSI) was added between the electrode and composite electrolytes to prevent the Ge reduction of LAGP via Li [57,58]. The CSP LAGP–LiTFSI DMF/H_2_O system exhibits lower area-specific resistance (ASR) (~1000 Ω cm^2^) than the CSP LAGP–LiTFSI H2O system (~1200 Ω cm^2^) (Figure S4a,c). Based on this, Figure 6 displays lithium plating–stripping behaviors, with the initial cycles presented in the inset. For the first 10 h, the cycle was carried out at 0.05 mA cm^−2^ for 1 h, after which, the current density was increased to 0.1 mA cm^−2^. At 0.05 mA cm^−2^, the CSP LAGP–LiTFSI H_2_O exhibited higher voltage profiles than CSP LAGP–LiTFSI DMF/H_2_O. However, when the current density was increased to 0.1 mA cm^−2^, the voltage profile of CSP LAGP–LiTFSI H_2_O suddenly dropped, indicating the presence of a short circuit. This was confirmed by the EIS result, which exhibited a very low resistance with an inductive loop, thus providing clear evidence to suggest that short circuiting occurred (Figure S4b) [59]. On the other hand, CSP LAGP–LiTFSI DMF/H_2_O exhibited a stable plating–stripping behavior, with no voltage drop over 350 h. These results suggest that composite electrolytes synthesized with H_2_O alone are unsuitable for use in Li metal batteries, especially when exposed to high temperatures. Our electrolytes comprise high ASR due to its thick nature, highlighting the need to develop CSP techniques that produce thin electrolytes with low ASR in the future.

## 4. Conclusions

In this study, we demonstrated the effectiveness of using a mixture of high b.p. DMF (or DMSO) and water to create thermally stable and highly conductive LAGP ceramic electrolytes via CSP. The DMF was found to have a high solvation ability for LiTFSI; therefore, the CSP LAGP–LiTFSI DMF/H_2_O mixture can form a stable DMF–LiTFSI phase in the grain boundary regions without any evaporation. In contrast, CSP LAGP–LiTFSI DMSO/H_2_O led to poor thermal stability due to DMSO’s limited solvation ability. As a result, the CSP LAGP–LiTFSI DMF/H_2_O electrolyte, at a ratio of 1:1, showed a high relative density of 87%, and a high σ of 1.65 × 10^−4^ S cm^−1^, with the σ remaining at 1.57 × 10^−4^ S cm^−1^ even after heat treatment at 60 °C for 24 h. Based on these performances, the Li symmetric cells fabricated after heat treatment exhibited a stable Li plating–stripping behavior over 350 h at 0.1 mAh cm^−2^. These results offer valuable insights into the optimization of CSP in terms of achieving stable and highly conductive oxide electrolytes, which could facilitate the development of cost-effective advanced energy storage systems.

## Data Availability

The data presented in this study are available on request from the corresponding author.

## Supplementary Materials

The following supporting information can be downloaded at: https://www.mdpi.com/article/10.3390/nano13172436/s1, Figure S1: Nyquist plot of (a) CSP LAGP–LiTFSI H_2_O, CSP LAGP–LiTFSI DMF/H_2_O, and CSP LAGP–LiTFSI DMSO/H_2_O electrolyte; (b) heat-treated CSP LAGP–LiTFSI H_2_O, CSP LAGP–LiTFSI DMF/H_2_O, and CSP LAGP–LiTFSI DMSO/H_2_O electrolytes. Figure S2: XRD patterns of LAGP powder and CSP LAGP–LiTFSI electrolytes with different processing solvents. Figure S3: ^1^H NMR spectra of (a) DMF/H_2_O–LiTFSI and (b) DMSO/H_2_O–LiTFSI with different H_2_O contents. Figure S4: Nyquist plot of the Li|CSP LAGP–LiTFSI H_2_O|Li cell (a) before and (b) after short circuiting. Li|CSP LAGP–LiTFSI DMF/H_2_O|Li cell (c) before and (d) after short circuiting. Table S1: Summary of relative densities and ionic conductivities studied in this work.
