# Supplementary material for: Thermally Stable Ceramic-Salt Electrolytes for Li Metal Batteries Produced from Cold Sintering Using DMF/Water Mixture Solvents
Source: Nanomaterials (Basel). 2023 Aug 28;13(17):2436. doi: 10.3390/nano13172436 (PMC10490499; doi:10.3390/nano13172436)
Supplement: Supplementary file 1 [file nanomaterials-13-02436-s001.zip › nanomaterials-2508289-supplementary.pdf]

nanomaterials

Supplementary Materials

Thermally Stable Ceramic-Salt Electrolytes for Li Metal Batteries Produced from Cold Sintering Using DMF/Water Mixture Solvents

Sunwoo Kim [1,2,†], Yejin Gim [1,2,†] and Wonho Lee [1,2,*]

[1] Department of Polymer Science and Engineering, Kumoh National Institute of Technology, Gumi 39177, Republic of Korea; rlatjsdn110@naver.com (S.K.); yj_gim@kumoh.ac.kr (Y.G.)
[2] Department of Energy Engineering Convergence, Kumoh National Institute of Technology, Gumi 39177, Republic of Korea
* Correspondence: 1holee@kumoh.ac.kr
† These authors contributed equally to this work.

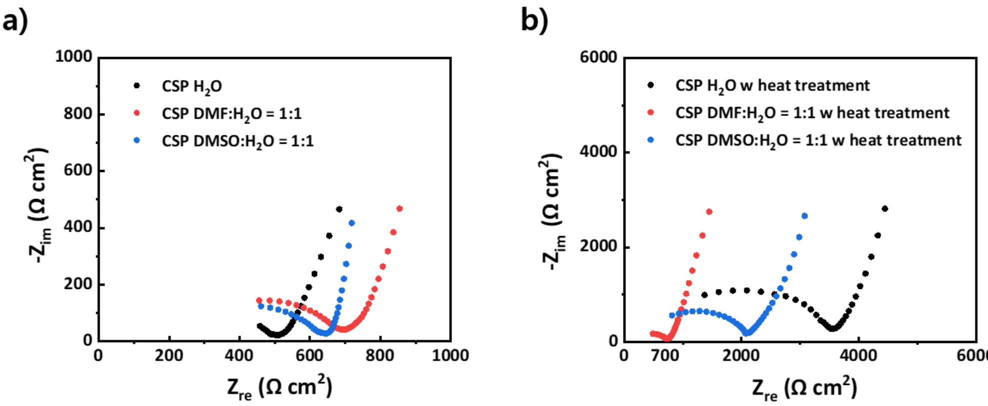

Figure S1. Nyquist plot of a) CSP LAGP-LiTFSI H_2O, CSP LAGP-LiTFSI DMF/H_2O, and CSP LAGP-LiTFSI DMSO/H_2O electrolyte; b) heat treated CSP LAGP-LiTFSI H_2O, CSP LAGP-LiTFSI DMF/H_2O, and CSP LAGP-LiTFSI DMSO/H_2O electrolyte.

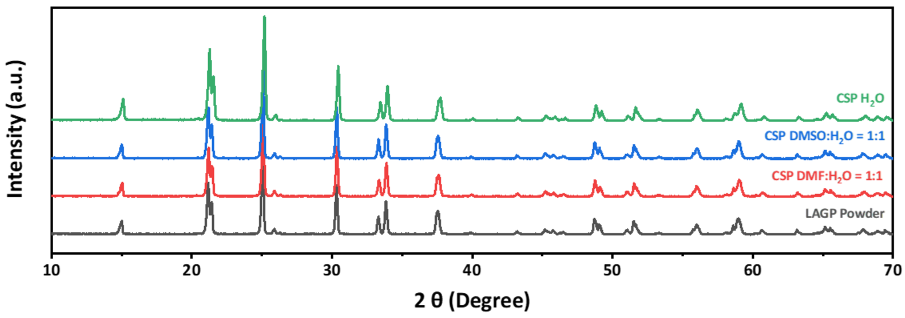

Figure S2. XRD patterns for LAGP powder and CSP LAGP-LiTFSI electrolytes with different processing solvents.

Citation: Kim, S.; Gim, Y.; Lee, W. Thermally Stable Ceramic-Salt Electrolytes for Li Metal Batteries Produced from Cold Sintering Using DMF/Water Mixture Solvents. *Nanomaterials* **2023**, *13*, x. https://doi.org/10.3390/xxxxx

Academic Editor(s): Zhan'ao Tan

Received: 30 June 2023
Revised: 1 August 2023
Accepted: 22 August 2023
Published: 25 August 2023

Copyright: © 2023 by the authors. Submitted for possible open access publication under the terms and conditions of the Creative Commons Attribution (CC BY) license (https://creativecommons.org/licenses/by/4.0/).

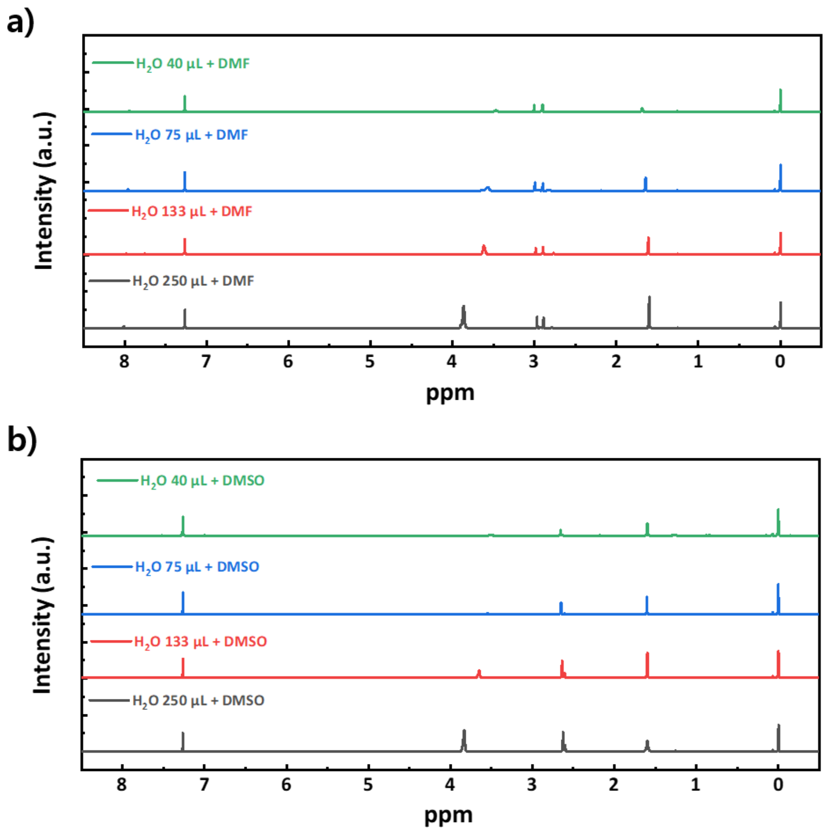

Figure S3. ^{1}H NMR spectra of a) DMF/H_2O-LiTFSI and b) DMSO/H_2O-LiTFSI with different H_2O contents.

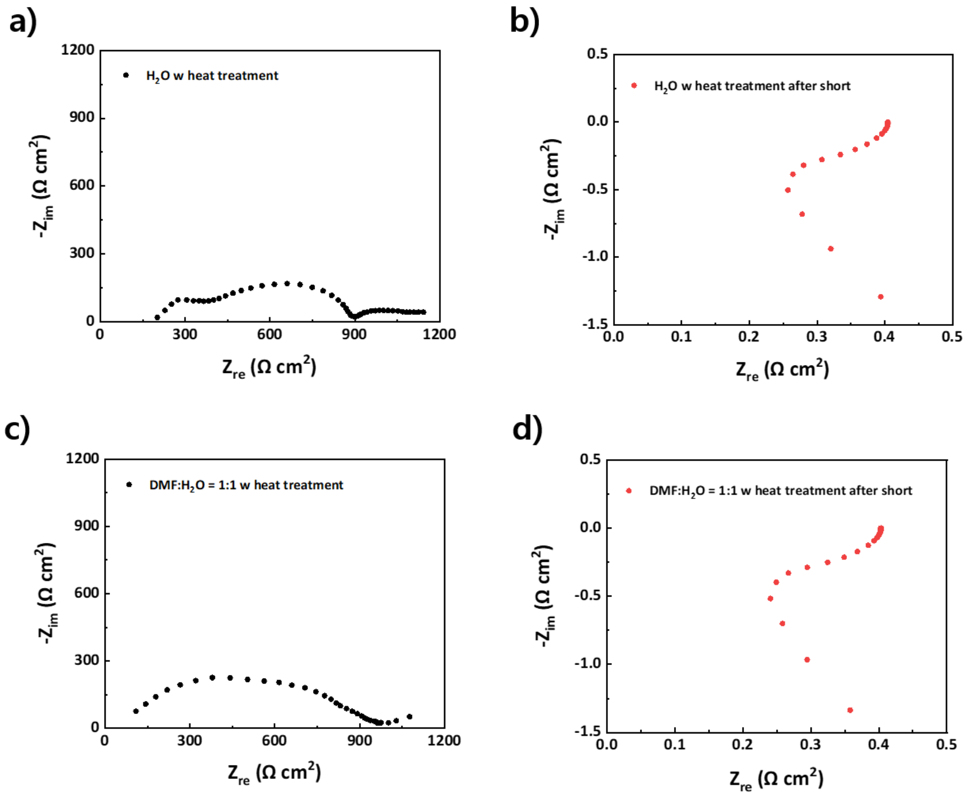

Figure S4. Nyquist plot of Li|CSP LAGP-LiTFSI H_2O|Li cell a) before and b) after short circuit. Li|CSP LAGP-LiTFSI DMF/H_2O|Li cell c) before and d) after short circuit.

Table S1. Summary of relative densities and ionic conductivities studied in this work. a) DMF:H_2O and (b) DMSO:H_2O

a)

DMF:H_2O	Relative density (%)	Ionic conductivity (S cm^{-1})	Ionic conductivity after annealed at 60 °C for 24 h (S cm^{-1})
0:1	91.0	2.50×10^{-4}	4.20×10^{-5}
1:9	90.0	1.55×10^{-4}	4.92×10^{-5}
1:4	89.2	2.48×10^{-4}	5.01×10^{-5}
3:7	87.7	2.30×10^{-4}	6.14×10^{-5}
2:3	89.8	2.43×10^{-4}	7.98×10^{-5}
1:1	87.2	1.65×10^{-4}	1.57×10^{-4}
3:2	85.3	1.15×10^{-4}	9.91×10^{-5}
7:3	83.0	4.30×10^{-5}	4.72×10^{-5}

b)

DMSO:H_2O	Relative density (%)	Ionic conductivity (S cm^{-1})	Ionic conductivity after annealed at 60 °C for 24 h (S cm^{-1})
0:1	91.0	2.50×10^{-4}	4.20×10^{-5}
1:9	88.2	2.73×10^{-4}	2.30×10^{-5}
1:4	86.4	2.30×10^{-4}	1.88×10^{-5}
3:7	90.0	1.16×10^{-4}	1.64×10^{-5}
2:3	86.2	1.71×10^{-4}	3.91×10^{-5}
1:1	88.8	1.76×10^{-4}	5.44×10^{-5}
3:2	87.8	1.25×10^{-4}	3.31×10^{-5}
7:3	82.6	9.36×10^{-5}	2.38×10^{-5}
